# Urinary incontinence as a possible signal of neuromuscular toxicity during immune checkpoint inhibitor treatment: Case report and retrospective pharmacovigilance study

**DOI:** 10.3389/fonc.2022.954468

**Published:** 2022-09-12

**Authors:** Yizhang Hu, Wenchao Lu, Borui Tang, Zhixia Zhao, Zhuoling An

**Affiliations:** ^1^ Department of Oncology, Beijing Chao-yang Hospital, Capital Medical University, Beijing, China; ^2^ Department of Pharmacy, Beijing Chaoyang Hospital, Capital Medical University, Beijing, China; ^3^ Department of Pharmacy Clinical Trial Research Center, China-Japan Friendship Hospital, Beijing, China

**Keywords:** urinary incontinence, immune-related adverse events, FAERS, neuromuscular adverse events, immune checkpoint inhibitors

## Abstract

**Background:**

Immune checkpoint inhibitors (ICIs) are associated with different immune-related adverse events (irAEs), but there is limited evidence regarding the association between urinary incontinence and ICIs.

**Methods:**

We described the case of a patient experiencing urinary incontinence who later experienced a series of irAEs such as myocarditis, myositis, and neurologic diseases while on ICI treatment in our hospital. In addition, we queried the Food and Drug Administration (FDA) Adverse Event Reporting System (FAERS) from the third quarter of 2010 to the third quarter of 2020 to perform a retrospective study to characterize the clinical features of urinary incontinence associated with ICIs.

**Result:**

In the FAERS study, 59 cases of ICI-related urinary incontinence were retrieved, and approximately 32.2% of the cases were fatal. Combination therapy with nervous system drugs and age >80 years old were the significant risk factors for fatal outcomes. Among these cases of ICI-related urinary incontinence, 40.7% (n = 24) occurred concomitantly with other adverse events, especially, neurological (fifteen cases), cardiovascular (seven cases), musculoskeletal (six cases), and urological disorders (five cases). Five cases had an overlapping syndrome similar to our case report, including one case of myasthenia gravis with myocarditis and another of myasthenic syndrome with polymyositis.

**Conclusion:**

ICI-related urinary incontinence might be a signal of fatal neuromuscular irAEs, especially when it occurs concomitantly with ICI-associated neuromuscular–cardiovascular syndrome. Clinicians should be aware of the occurrence of urinary incontinence to identify potentially lethal irAEs in the early phase.

## Introduction

Immune checkpoint inhibitors (ICIs) have revolutionized cancer therapy and improved clinical outcomes in multiple cancer types (1). To date, approved ICIs include anti-cytotoxic T-lymphocyte-associated antigen 4 (CTLA-4; ipilimumab), anti-programmed cell death-protein-1 (PD-1; pembrolizumab, nivolumab, cemiplimab), and anti-programmed cell death-ligand 1 (PD-L1) therapies (atezolizumab, durvalumab, avelumab) (2). Despite their important clinical benefits, ICIs cause a unique spectrum of side effects termed immune-related adverse events (irAEs). These events can affect many organ systems, and they can be fulminant or even fatal in some cases (3).

During routine surveillance, we identified a patient with unusual urinary incontinence symptoms during immunotherapy treatment. In addition, urinary incontinence did not develop as an isolated adverse effect. A more detailed medical examination revealed that the patient experienced a series of irAEs such as myocarditis, myositis, and neurological diseases. Urinary incontinence is the involuntary leakage of urine (4), and its pathogenic causes include neuromuscular diseases, inflammation, or infection of the bladder or urethral wall and bladder outlet obstruction (5). In addition, the neural control of the lower urinary tract and pelvic floor musculature is essential for urine storage. Therefore, the damage of these areas is one of the leading causes of urinary incontinence (5). ICIs can damage the function of nerves and muscles *via* lymphocyte-rich infiltration, antibody-mediated inflammation, and sterile inflammation (6). No study has demonstrated an association between urinary incontinence and ICI treatment. Conversely, ICI-related neuromuscular adverse events, such as ICI-related myelitis, Guillain–Barré syndrome (GBS), and myasthenia gravis–myositis syndrome, were reported to be linked to urinary incontinence symptoms (7–9). This suggests that urinary incontinence is secondary to irAEs.

To date, there has been limited research investigating the association between ICIs and urinary incontinence. We only identified one similar case report from the published literature (7). Recently, the FDA Adverse Event Reporting System (FAERS) database has been increasingly utilized to quickly detect rare and unexpected adverse events. Therefore, we reviewed the reported cases of urinary incontinence after ICI treatment and described the concomitant irAEs and characteristics by retrospectively analyzing the FAERS database.

## Case report

A 65-year-old woman was diagnosed with clinical stage IIIC (cT3N3M0) pulmonary adenocarcinoma 2 months prior to hospitalization with no actionable somatic mutation and a tumoral cell PD-L1 status of ≥50%. She had opted to join a clinical trial program that included immunotherapy, anti-vascular endothelial growth factor (VEGF) therapy, and chemotherapy. She received two cycles of treatment consisting of the anti-PD-1 antibody (HLX10 4.5 mg/kg every 3 weeks), anti-VEGF monoclonal antibody (HLX04 bevacizumab biosimilar, 15 mg/kg every 3 weeks), and carboplatin (area under the concentration–time curve = 5 mg/ml/min) plus pemetrexed (500 mg/m^2^). The initial examination was unremarkable. After the first cycle of treatment, she experienced a liver injury. After oral hepatic protectants (bicyclol, polyene phosphatidylcholine capsules) were used, her symptoms significantly improved.

During the two-cycle treatment, the patient displayed sudden urinary incontinence after her body position changed. The severity of urinary incontinence required the patient to use sanitary napkins to move about in the ward. Subsequently, the patient exhibited mild fatigue. The neurological examination was unremarkable, and no evidence of tenderness to the palpation of major muscle groups, decreased muscle strength, or ptosis were noted. Notable laboratory abnormalities are listed in **Supplementary Table 1**.

Electrocardiogram (ECG) demonstrated a normal sinus rhythm with a slight decrease in the R wave amplitude. Echocardiography, cardiac magnetic resonance, brain magnetic resonance imaging, abdominal and pelvic computed tomography (CT), and kidney/bladder ultrasound revealed normal findings. Chest CT revealed increased pericardial thickness. Needle electromyography uncovered left external anal sphincter neurogenic impairment (**Supplementary Table 2**). In addition, nerve conduction studies revealed peripheral neurogenic impairment, which may have involved the motor nerves with demyelination (**Supplementary Table 3**). This finding is consistent with previous clinical observations that the principal manifestation of ICI-related neuropathy is motor nerve demyelination (10). A urodynamic study (UDS) found a reduction in intraurethral pressure. A repetitive nerve stimulation study suggested the abnormality of the neuromuscular junction (**Supplementary Table 4**). Notably, AChR antibody testing was positive. These results supported a diagnosis of myasthenia gravis or Lambert–Eaton myasthenic syndrome. Additionally, based on her elevated creatine kinase (CK), myocardial enzyme, and cardiac troponin I (cTnI) and the changes of ECG data, she was suspected to have myositis and myocarditis. All of these findings suggested that the patient had developed an overlap of urinary incontinence–myasthenia gravis–neuropathy–myositis–myocarditis-like syndrome.

**Figure 1:**
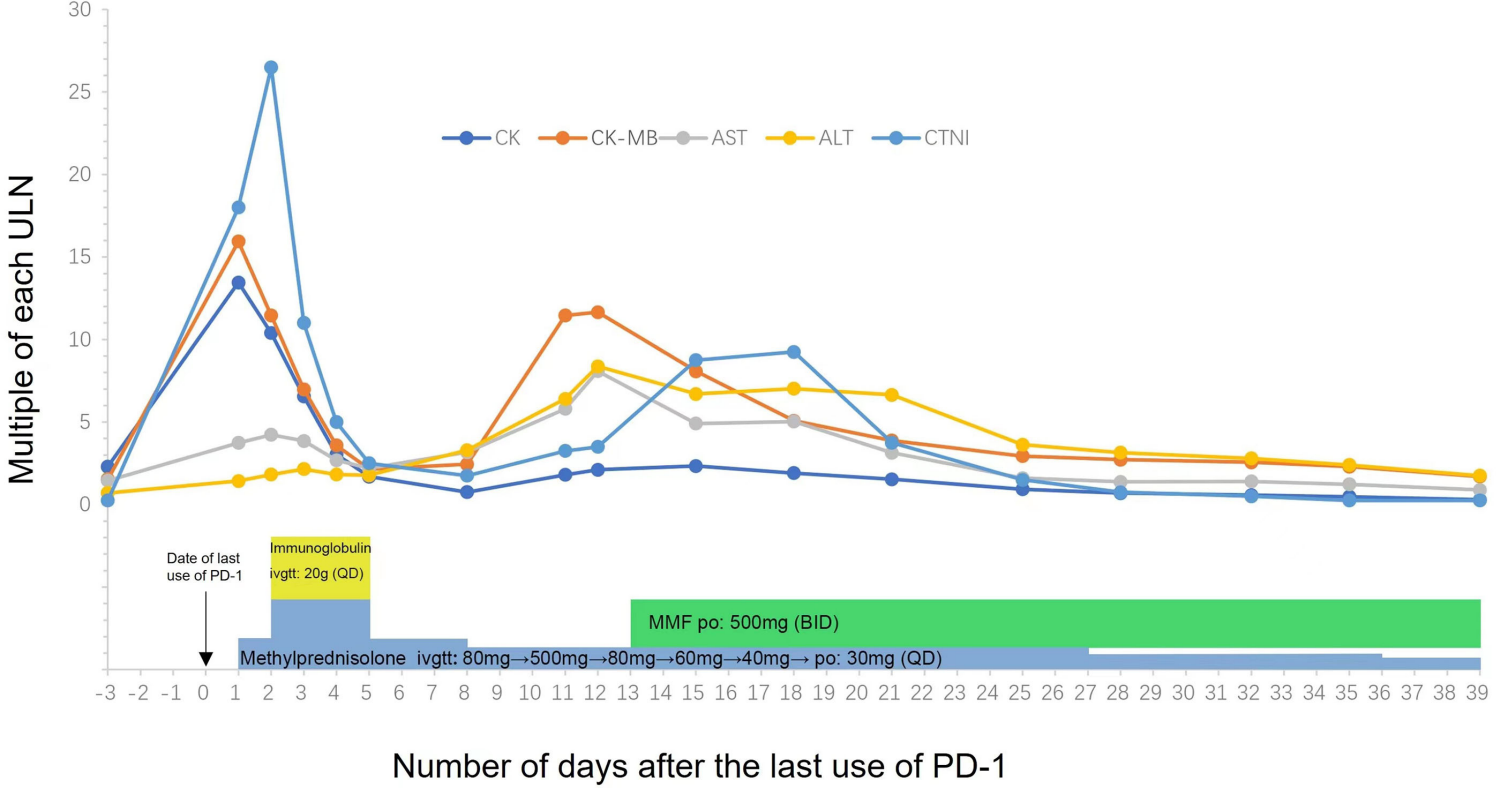
Temporal changes of creatinine kinase (CK), cardiac troponin I (cTnI) and myocardial enzyme (AST/ALT/CK-MB). Treatments performed are indicated below the graph. MMF, Mycophenolate Mofetil.

Then, the patient was administered intravenous methylprednisolone 80 mg/day for 2 days. Her CK and CK-MB isoenzyme (CK-MB) levels were decreased slightly by treatment, but her cTnI levels increased rapidly. Subsequently, the patient received steroid pulse therapy (methylprednisolone 500 mg) and intravenous immune globulin 40 mg/kg/day for 3 days. Her symptom of incontinence improved, and her CK, CK-MB, and cTnI levels were decreased by this treatment (**Figure 1**). During the process of glucocorticoid tapering (from 80 to 40 mg/day), her myocardial enzyme and cTnI levels rose again (**Figure 1**), and the patient developed new-onset hoarseness. Mycophenolate mofetil (MMF) 1,000 mg/day was thus prescribed. Following this treatment, the patient’s CK-MB and cTnI gradually decreased to normal, and urinary incontinence was relieved. MMF and corticosteroids were discontinued at 11 and 14 weeks following symptom onset, respectively. The radiographic evaluation after two cycles of treatment revealed partial tumor remission. Six months after the last antitumor treatment, retreatment with the original chemotherapy regimen (carboplatin plus pemetrexed) combined with bevacizumab was performed because of lesion progression. The tumor was effectively controlled again, and urinary incontinence did not recur.

Overall, we suspected that urinary incontinence syndrome was caused by ICI therapy for several reasons. First, there was a temporal association between ICI treatment and the occurrence of urinary incontinence. Second, the patient underwent urinalysis, urine culture, ultrasound, and UDS, and the results did not uncover other potential causes of urinary incontinence. Third, the patient was retreated with the original chemotherapy regimen (carboplatin plus pemetrexed) combined with bevacizumab, and urinary incontinence did not recur.

However, the electrodiagnostic data and clinical manifestations of neuromuscular toxicity were atypical in this case, which could potentially have several explanations. First, we identified the disease in an extremely early stage, and neither the patient’s symptoms nor the electrodiagnostic data were typical at this stage. Second, the patient was treated with high-dose steroids and intravenous immunoglobulin in an extremely early stage; therefore, the disease was relieved to a certain extent before electrodiagnostic testing. Finally, the patient had overlapping neuromuscular toxicity, and different diseases interacted with each other, which might have affected the electrodiagnostic data.

## Descriptive analysis based on the FDA adverse event reporting system database

### Definition and design

We downloaded FAERS data files from the third quarter of 2010 to the third quarter of 2020. We used generic and brand names to identify drugs, including anti-CTLA-4 (ipilimumab), anti-PD-1 (pembrolizumab, nivolumab, cemiplimab), and anti-PD-L1 therapies (atezolizumab, avelumab, durvalumab). We identified cases of urinary incontinence using the preferred term “urinary incontinence” according to the Medical Dictionary for Regulatory Activities (version 23.0). Duplicate reports were excluded from our analysis. In the deduplication process, we extracted the latest (most recent) case version from all available cases based on the case ID, case initial/follow-up code (“I” or “F”), case event date, age, sex, and reporting country (11). We retained the most current case version and removed all others.

To summarize the clinical characteristics of cases of ICI-related urinary incontinence, we analyzed general information, patient characteristics, indications for ICIs, outcomes (serious events defined as death, hospitalization, life-threatening events, or disability), the role of ICIs (primary suspected, secondary suspected, and concomitant), and ICI treatment strategy (monotherapy or combination therapy). Additionally, we analyzed the drugs used concomitantly with ICIs, such as nervous system drugs (including benzodiazepines, antipsychotics, antiepileptics, and antidepressants), α-adrenoceptor antagonists, and diuretics, which have been reported to increase the risk of urinary incontinence (12). We further analyzed the concurrent adverse events (AEs), especially neuromuscular toxicity and cardiotoxicity, which also occurred in our case. Clinical characteristics were described using quantities and proportions for qualitative variables and medians (with the interquartile range) for quantitative variables. We also performed a subgroup analysis to explore the differences in the clinical characteristics of severe and non-severe ICI-associated urinary incontinence. Proportions were compared using Pearson’s chi-square or Fisher’s exact test. Data were analyzed using SPSS (v22.0; IBM Corp, Armonk, NY, USA), and statistical significance was indicated by p < 0.05.

### Results of the FDA adverse event reporting system analysis

Our analysis of the FAERS database captured 96,814 AEs related to ICI treatment, including 59 cases of urinary incontinence (45, 5, 2, and 7 events associated with anti-PD-1, anti-PD-L1, anti-CTLA-4, and combination therapies, respectively). **Table 1** presents the characteristics of patients with ICI-related urinary incontinence. Most cases of ICI-related urinary incontinence occurred in patients with lung cancer and melanoma (35.6% and 23.7%, respectively), and most cases were reported in the Americas (40 [67.8%]). The mean age of the affected patients was 70.0 ± 11.3 years. Meanwhile, patients older than 80 years were more likely to experience fatal outcomes (**Table 2**, Fisher’s exact test, p = 0.003). Urinary incontinence was more common in men than in women (54.2% versus 44.1%). The results of time-to-onset (TTO) analysis for urinary incontinence associated with ICI are also summarized in **Table 1**. The median TTO was 16 days (interquartile range = 6–82), suggesting that urinary incontinence most commonly occurred in the early period.

**Table 1:** The characteristics of patients with immune checkpoint inhibitor (ICI)–related urinary incontinence.

Characteristics	n (%)	Characteristics	n (%)
**Total AE (ICIs)**	96814	**ICI Treatment Strategy**	
Total urinary incontinence	59	**Monotherapy**	
**Occurred Region**		PD-1 inhibitor	45 (76.3)
Americas	40 (67.8)	Nivolumab	23 (39.0)
Europe	9 (15.2)	Pembrolizumab	22 (37.3)
Asia	4 (6.8)	PD-L1 inhibitor	5 (8.5)
Others	6 (10.2)	Durvalumab	3 (5.1)
**Reporting Year**		Avelumab	1 (1.7)
2020	13 (22.0)	Atezolizumab	1 (1.7)
2019	8 (13.6)	CTLA-4 inhibitor	
2018	17 (28.8)	Ipilimumab	2 (3.4)
2017	13 (22.0)	**Combination Therapy**	
2016 and earlier	8 (13.6)	Ipilimumab + nivolumab	7 (11.9)
**Reporter Type**		**Concurrent Drugs**	14(23.7)
Healthcare professional	37 (62.7)	**Single Concurrent Drugs**	8(13.5)
Non-healthcare professional	21 (35.6)	α-Adrenoceptor antagonists	3 (5.1)
Null	1 (1.7)	Nervous system drug	4 (6.7)
**Gender**		Diuretics	1 (1.7)
Male	32 (54.2)	**Multiple Concurrent Drugs**	6(10.2)
Female	26 (44.1)	α-Adrenoceptor antagonists +	1 (1.7)
Null	1 (1.7)	Nervous system drug	
**Age (n=49)**		Multiple nervous system drugs	3 (5.1)
Mean, SD	70.0 ± 11.30	Diuretics +	2 (3.4)
Range	41-87	Nervous system drug	
**Indication for ICI**		**Concurrent AEs**	24 (40.7)
Lung cancer	21 (35.6)	**Neurological AEs**	15
Melanoma	14 (23.7)	Encephalitis	4 (6.8)
Renal cancer	6 (10.2)	Myasthenia gravis/	
Glioblastoma	3 (5.1)	Myasthenic syndrome	4 (6.8)
Breast cancer	2 (3.4)	Facial paralysis	3 (5.1)
Ovarian cancer	2 (3.4)	Neuropathy	2 (3.4)
Cholangiocarcinoma	2 (3.4)	Encephalopathy	2 (3.4)
Pancreatic carcinoma	1(1.7)	Nervous system disorder	1 (1.7)
Colon cancer	1(1.7)	**Cardiovascular AEs**	7
Skin cancer	1(1.7)	Myocarditis	2 (3.4)
Acute myeloid leukemia	1(1.7)	Pericardial effusion	2 (3.4)
Transitional cell carcinoma	1(1.7)	Atrial fibrillation	1(1.7)
Prostate cancer	1(1.7)	Cardiac arrest	1 (1.7)
Bladder cancer	1(1.7)	Cardiac disorder	2 (3.4)
Others	2(3.4)	**Musculoskeletal AEs**	6
**Outcomes**		Immune-mediated myositis	1 (1.7)
Death	19 (32.2)	Polymyositis	1 (1.7)
Life-Threatening	4 (6.8)	Arthritis	1 (1.7)
Other serious	17 (28.8)	Musculoskeletal disorder	3 (5.1)
Hospitalization	12 (20.3)	**Urinary AEs**	5 (8.5)
Null	7 (11.9)	Cystitis	1 (1.7)
**ICIs Role**		Acute kidney injury	1 (1.7)
Primary suspected	48 (81.4)	Renal disorder	3 (5.1)
Secondary suspected	8 (13.6)	Bladder disorder	1 (1.7)
Concomitant	3 (5.1)	**Other AEs**	13 (22.0)
**Suspected Drugs**		**Time to onset (days)**	
Only ICI	41 (69.5)	Median (IQR)	16 (6-82)
ICI plus one other drug	7 (11.9)		
ICI plus two or more other	8 (13.6)		
drugs			
Only other drugs	3 (5.1)		

**Table 2:** Differences in clinical characteristics of fatal and non-fatal ICI-associated urinary incontinence cases.

		Fatal cases	Non-fatal cases	P value	
**Total**		19	40		
**Gender**	Male	9	23	0.465	a
	Female	9	17	0.725	a
	Unknown	1		0.322	b
**Age**	Median(n=49)	73 (n=18)	67.5 (n=31)		
	<60	4	8	1.000	b
	60–80	7	21	0.260	a
	>80	7	2	0.003	b
**Indications**	Lung cancer	7	14	0.890	a
	Melanoma	5	9	0.753	b
	Renal cancer	2	4	1.000	b
	Glioblastoma	0	3	0.544	b
	Breast cancer	0	2	1.000	b
	Ovarian cancer	1	1	0.544	b
	Cholangiocarcinoma	1	1	0.544	b
	Pancreatic carcinoma	0	1	1.000	b
	Colon cancer	1	0	0.322	b
	Skin cancer	0	1	1.000	b
	Acute myeloid Leukemia	0	1	1.000	b
	Bladder cancer	1	0	0.322	b
	Transitional cell carcinoma	0	1	1.000	b
	Prostate cancer	1	0	0.322	b
	Others	0	2	1.000	b
**ICI drugs**	PD-1	14	31	0.753	b
	PD-L1	2	3	0.653	b
	CTLA-4	1	1	0.544	b
	PD-1+CTLA-4	2	5	1.000	b
**Concurrent Drugs**	Nervous system drug	8	3	0.003	b
	α-Adrenoceptor antagonists	1	3	1.000	b
	Diuretics	2	1	0.240	b
**Concurrent AE**	Neurological AEs	5	10	1.000	b
	Myasthenia gravis/Myasthenic syndrome	3	1	0.094	b
	Cardiovascular AEs	3	4	0.670	b
	Myocarditis	1	1	0.544	b
	Musculoskeletal AEs	3	3	0.376	b
	Myositis	2	0	0.100	b
	Urinary AEs	2	2	0.588	b
	Other AEs (dermatologic. pulmonary, endocrine, gastrointestinal)	3	10	0.517	b
	Overlapping	5	11	0.924	a

a: Pearson χ2 test.

b: Fisher’s exact test.

Our study identified patients with urinary incontinence who experienced poor outcomes. Approximately 32.2% of these events resulted in death, 6.8% were life-threatening, and 20.3% led to hospitalization. Most patients with ICI-related urinary incontinence were treated with polypharmacy. In total, 23.7% (14/59) of cases involved the concurrent use of drugs influencing bladder function, and the most common concurrent drugs were nervous system drugs (benzodiazepines, antipsychotics, antidepressants, antiepileptic), followed by α-adrenoceptor antagonists and diuretics. In cases involving concurrent nervous system drug use, the fatality rate was even higher, at 72.7% (8/11). In an analysis of fatal versus non-fatal cases (**Table 2**), we confirmed that patients who concurrently used nervous system drugs were more likely to have fatal outcomes (Fisher’s exact test, p = 0.003).

Considering the possible confounding factors, we also checked the overall anticancer regimen in our case and other risk factors of urinary incontinence recorded in the FAERS database. In total, 16.9% (10/59) of the cases were treated with combination therapy featuring vascular endothelial growth factor receptor (VEGFR) inhibitors and/or chemotherapy. Specifically, 10.2% (6/59) were treated with chemotherapy, 5% (3/59) were treated with VEGFR inhibitors, and only 1.7% (1/59) were treated with both drugs.

Among the cases of ICI-related urinary incontinence, 39.0% (n = 23) occurred concurrently with other AEs, especially neurological disorders (n = 15 [25.4%]) such as myasthenia gravis (three cases) and myasthenic syndrome (one case). In addition, the most commonly reported concurrent symptoms were neuromuscular systems, including fatigue, muscular weakness, ptosis, hoarseness, and dysphagia, which were found in 86.4% (51/59) of the cases.

Similar to the aforementioned case report, in the FAERS database, we also found five cases of ICI-associated neuromuscular–cardiovascular overlapping syndrome. The neuromuscular AEs mainly included myasthenia gravis, myasthenic syndrome, and myositis. Case 5 was not diagnosed with myasthenia gravis/myasthenic syndrome, but ptosis was the specific symptom. The cardiovascular disorders mainly included myocarditis, cardiac arrhythmias, and pericardial effusion. In addition, the prognosis was poor, and the outcomes included three fatalities and two hospitalizations. **Table 3** presents the descriptive characteristics of these reports.

**Table 3:** ICI-related urinary continence concomitant with neuromuscular–cardiovascular syndrome case series in the FDA Adverse Event Reporting System database.

Patient	Age	Gender	Indication	ICIs	Concurrent AEs	Outcome
**1**	62	F	Non-small cell lung cancer	Nivolumab	Myasthenia gravis, facial paralysis, hepatic enzyme increased	Death
**2**	73	M	Malignant melanoma	Pembrolizumab	Myasthenia gravis, atrial fibrillation	Hospital
**3**	54	M	Cholangiocarcinoma	Nivolumab	Myasthenia gravis, myocarditis	Death
**4**	71	M	Non-small cell lung cancer	Pembrolizumab	Myasthenic syndrome, pericardial effusion, Bradycardia, heart injury, Polymyositis	Death
**5**	54	M	Cholangiocarcinoma	Nivolumab	Myocarditis, eyelid ptosis	Hospital

## Discussion

In our study, we reported a case of urinary incontinence after ICI treatment in a patient with non-small cell lung cancer who also had other irAEs, including myasthenia gravis, neuropathy, myositis, and myocarditis. Then, we reviewed the FAERS database to analyze more cases of ICI-related urinary incontinence, summarized the clinical characteristics, and explored the correlation between urinary incontinence and other ICI-related disorders.

In the published literature, limited research has examined the association between ICIs and urinary incontinence. One study reported a case similar to ours, as it described a patient who developed an overlap syndrome consisting of myasthenia gravis, myositis, and myocarditis after cancer immunotherapy, and the patient also experienced urinary and fecal incontinence. In addition, there were some common characteristics among the cases reported by Ng et al. and our group, such as the early onset of AEs and poor prognoses (7).

### Possible mechanisms

The mechanism of ICI-associated urinary incontinence remains unclear. Generally, urinary incontinence is usually caused by neuromuscular disorders that influence urinary storage and voiding. Additionally, some urological diseases such as cystitis can induce urinary incontinence. In our retrospective study of the FAERS database, it was evident that urinary incontinence does not develop as an isolated adverse effect, as it always occurred concomitantly with neuromuscular irAEs.

ICIs can cause nerve and muscle damage through lymphocyte infiltration, antibody-mediated inflammation, and sterile inflammation (13). In addition, irAEs can present as central nervous system (CNS) diseases (such as aseptic meningitis, encephalitis, CNS demyelinating diseases, and transverse myelitis), peripheral nervous system diseases (such as peripheral neuropathy, GBS, myasthenia gravis, and Lambert–Eaton myasthenic syndrome), and myositis (13–15). Several studies reported that ICI-related neuromuscular disorders induced urinary incontinence. For example, sphincter dysfunction occurs in 86%–92% of patients with ICI-related myelitis, and the related symptoms include urinary incontinence (9). Bladder dysfunction was observed in patients with GBS at rates ranging from 25% to more than 80% (16), and the symptom could present as urinary incontinence (17, 18). Kelly et al. reported that ipilimumab could induce GBS, and their patient presented with dysautonomia that manifested as urinary retention (8). Meanwhile, other neuromuscular irAEs could theoretically cause lower urinary tract dysfunction based on the characteristics of the primary disease. For example, autoimmune encephalitis could present with bladder dysfunction (19, 20). Diabetic peripheral neuropathy may be associated with urinary incontinence, which manifested as urge incontinence (5). In total, 11.7%–72% of patients with multiple sclerosis developed urinary incontinence (21–25). Myasthenia gravis can predispose individuals to a higher risk of urinary incontinence by affecting the tone of the smooth or striated muscle of the distal sphincter (5). The frequency of urinary incontinence was significantly higher in patients with myasthenia gravis than in controls (26, 27). Sandler et al. concluded that voiding dysfunction heralded either a new diagnosis of myasthenia gravis or an exacerbation of the disease process (28). The Lambert–Eaton myasthenic syndrome is characterized by autonomic dysfunction, which is also experienced as voiding dysfunction in some cases (29). Inflammatory myopathies can also affect bladder/urinary function by decreasing pelvic floor function (30).

Moreover, it is well known that in patients with systemic autoimmune diseases (such as systemic lupus erythematosus, Sjögren’s syndrome, and rheumatoid arthritis), non-bacterial cystitis can develop and, in turn, contribute to urinary incontinence. In one study, bladder biopsy samples displayed lymphocyte infiltration and increased numbers of mast cells (31). Consistently, there were cases featuring coincident cystitis in our FAERS database analysis. Several studies reported that ICI-related non-bacterial cystitis could also induce bladder dysfunction (32–35). Meanwhile, the analyses of bladder biopsy samples also revealed numerous events of lymphocyte infiltration into the urothelium (32, 35).

### Concomitant adverse events

Neuromuscular irAEs were the most frequently reported concomitant AEs with urinary incontinence. Concomitant neurological AEs included encephalitis, encephalopathy, facial paralysis, neuropathy, and myasthenia gravis/myasthenic syndrome. Concomitant musculoskeletal irAEs included two cases of myositis. In addition, concomitant urological irAEs included one case of cystitis. Myelitis, multiple sclerosis, and GBS are common neurological causes of bladder dysfunction. The related concomitant AEs were not observed in the FAERS database. However, the typical symptoms of these diseases were reported (including paraparesis, paresthesia, muscular weakness, diplopia, seizure, and ataxia). This suggests that many neurological irAEs might be underreported. This might be attributable to their non-specific symptoms, their low incidence, and a lack of recognition of these irAEs among oncologists (36, 37).

### Other interferences

We additionally reviewed anticancer regimens reported in the FAERS database. Combined treatment with other anticancer therapies was only reported in limited cases. In our case, the patient was rechallenged with the original chemotherapy regimen combined with VEGFR inhibitors, and urinary incontinence did not recur. We also explore the related literature in PubMed and EMBASE. There is no evidence demonstrating that VEGFR inhibitors can induce urinary incontinence. Regarding chemotherapy, carboplatin/paclitaxel therapy for gynecologic cancers may lead to new-onset or worsening urinary incontinence, most likely related to paclitaxel (38). However, the use of paclitaxel for gynecologic cancers was rare in our findings. Overall, these results suggest that urinary incontinence is mainly relevant to ICIs.

### Prognosis and mortality

In this FAERS study, patients with urinary incontinence experienced relatively severe outcomes, especially a high mortality rate. However, urinary incontinence is not inherently dangerous. Therefore, we must realize that death is not necessarily related to the drug/events, but it could possibly be related to the underlying disease. Because of the limitation of the FAERS database, we could not obtain information on the underlying illnesses of patients. By analyzing the concomitant drugs, we found that nervous system drugs were the most common concomitant drugs, which means that patients with urinary incontinence may also have nervous system disease. Notably, patients who used nervous system drugs had a significantly higher risk of fatal outcomes in the analysis (**Table 2**). Furthermore, ICI-related urine incontinence always occurs concomitantly with neuromuscular irAEs or neuromuscular–cardiovascular overlapping syndrome, both of which have high risks of mortality and are difficult to manage (13, 39, 40). Therefore, further investigation into the prognosis and mortality of ICI-related urine incontinence is needed.

### Management suggestions

The occurrence of urinary incontinence during ICI treatment points toward possible life-threatening neuromuscular AEs, which should be assessed using relevant tests. For example, muscle and myocardial enzyme and cTn levels should be tested. Electrophysiology, neuroimaging, lumbar puncture, and antineuronal/AChR antibody measurements should be used to identify ICI-induced nervous systems toxicity. UDS, urological ultrasound, urinalysis, and urine culture should be performed to identify other urinary system diseases. In addition, ECG should also be performed to identify ICI-induced myocarditis and myositis. The most important thing is to be aware of potentially lethal neuromuscular irAEs based on the presence of urinary incontinence, muscle weakness, fatigue, myalgia, or dyspnea. In addition, proactive and effective treatments are also crucial. Glucocorticoids represent the mainstay of treatment; our case appeared to involve steroid-dependent irAEs. Additional immunosuppressant and intravenous immunoglobulin therapy effectively improved the disease. This was consistent with the disease characteristics of the ICI-associated overlap syndrome in previous studies and the aforementioned similar case (40). These findings remind us that ICI-related urinary incontinence might require intensive monitoring and combination therapy.

### Limitations

We acknowledge that our study had limitations. We were unable to calculate the incidence because FAERS lacks denominators, and it does not receive reports for every AE that occurs with a product. Additionally, the lack of test data and clinical elements, such as laboratory data, some radiological findings, and preexisting disease, makes it challenging to fully analyze all of the confounders involved in the occurrence of AEs. Notwithstanding these limitations, information within FAERS can support the data or information found in clinical research or published studies. In some cases, FAERS data can provide meaningful postmarketing signals of rare AEs not observed in clinical trials.

## Conclusion

ICI-related urinary incontinence might represent a signal of neuromuscular irAEs, which are associated with poor prognoses. Among the cases of urinary incontinence featuring concomitant irAEs, it is essential to remain vigilant regarding neuromuscular toxicities, especially myasthenia gravis–myocarditis–myositis syndrome, which has a high fatality rate. In addition, patients who received combination treatment with nervous system drugs or those of age > 80 years might have a higher risk of fatal outcomes. The early detection and engagement of a multidisciplinary team are critical, and high-dose glucocorticoid/immunomodulator therapy should be implemented.

## Data availability statement

The datasets presented in this study can be found in online repositories. The names of the repository/repositories and accession number(s) can be found below: https://www.fda.gov/drugs/questions-and-answers-fdas-adverse-event-reporting-system-faers/fda-adverse-event-reporting-system-faers-latest-quarterly-data-files.

## Ethics statement

Ethical review and approval was not required for the study on human participants in accordance with the local legislation and institutional requirements. The patients/participants provided their written informed consent to participate in this study. Written informed consent was obtained from the individual(s) for the publication of any potentially identifiable images or data included in this article.

## Authors contributions

YH, WL, ZZ, and ZA designed the study and developed the protocol. WL supported programming and prepared the data. YH provided the case report materials. BT did the statistical analysis. YH and WL wrote the first draft of the manuscript. WL, YH, ZZ, and ZA were involved in data review. All authors contributed to the critical revision of the manuscript for important intellectual content and approved the final version of the manuscript.

## Conflict of interest

The authors declare that the research was conducted in the absence of any commercial or financial relationships that could be construed as a potential conflict of interest.

## Publisher’s note

All claims expressed in this article are solely those of the authors and do not necessarily represent those of their affiliated organizations, or those of the publisher, the editors and the reviewers. Any product that may be evaluated in this article, or claim that may be made by its manufacturer, is not guaranteed or endorsed by the publisher.
