# Supplementary material for: Urinary incontinence as a possible signal of neuromuscular toxicity during immune checkpoint inhibitor treatment: Case report and retrospective pharmacovigilance study
Source: Front Oncol. 2022 Sep 12;12:954468. doi: 10.3389/fonc.2022.954468 (PMC9510979; doi:10.3389/fonc.2022.954468)
Supplement: Supplementary file 1 [file DataSheet_1.docx]

# **Supplementary Table 1: The highestc laboratory abnormalities and results of autoimmune testing.**

| **The highest laboratory abnormalities** | **Recorded values** | **Normal values** |
| --- | --- | --- |
| Creatine kinase (CK) | 2691 U/L | 40-200 U/L |
| Cardiac troponin I(cTnI) | 1.06 ng/ml | ≤0.04 ng/ml |
| creatine kinase MB(CK-MB) | 79.9 ng/mL | ≤5 ng/mL |
| Brain natriuretic peptide (BNP) | 276 pg/ml | ≤100 |
| Aspartate aminotransferase(AST) | 283 U/L | 13-35 U/L |
| Alanine aminotransferase(ALT) | 335 U/L | 7-40 U/L |
| ***Autoimmune antibody testing*** |  |  |
| Acetylcholine receptor antibody levels | 3.75 nmol/L | ＜0.45 nmol/L |

**Supplementary Table 2:** **Electromyography study**

| **Muscle** | **Insertional Activity** | **Fibrillation** | **Positive Sharp Wave** | **Fasciculation** | **Satellite potential** | **Duration** | **Amplitude** | **Polyphasic wave** | **Recruitment** |
| --- | --- | --- | --- | --- | --- | --- | --- | --- | --- |
| left external anal sphincter | Normal | None | None | None | >10% | prolonged 29% | Normal | 47% | reduced |

* Deltoid, vastus medialis muscles were tested and found to be normal.

**Supplementary Table 3: Nerve conduction study**

| **Nerve and site** | **Latency** | **Amplitude** | **Conduction velocity** | **F-wave latency** | **F-waves chronodispersion** |
| --- | --- | --- | --- | --- | --- |
| Motor conduction |  |  |  |  |  |
| Left tibialis | prolonged | normal | normal | normal | abnormal |
| Right tibialis | prolonged | low | block | prolonged | abnormal |
| Left peroneus | normal | normal | normal | absent | normal |
| Right peroneus | normal | normal | normal | normal | abnormal |

* Sensory nerve conduction was tested and found to be normal.

**Supplementary Table 4:** **Repetitive nerve stimulation study**

| **Type of stimuli** | **3 hertz** | **5** **hertz** | **30 hertz** | **50 hertz** |
| --- | --- | --- | --- | --- |
| Never |  |  |  |  |
| Right axillary nerve | No decrement | No decrement | decrement maximum -41% | / |
| Right facial nerve | No decrement | No decrement | increment maximum 102% | / |
| Right radial nerve | No decrement | No decrement | increment maximum 83% | increment maximum 107% |
